# Serum CXCL9 Levels Are Associated with Tumor Progression and Treatment Outcome in Patients with Nasopharyngeal Carcinoma

**DOI:** 10.1371/journal.pone.0080052

**Published:** 2013-11-21

**Authors:** Li-Jen Hsin, Huang-Kai Kao, I-How Chen, Ngan-Ming Tsang, Cheng-Lung Hsu, Shiau-Chin Liu, Yu-Sun Chang, Kai-Ping Chang

**Affiliations:** 1 Department of Otolaryngology-Head & Neck Surgery, Chang Gung Memorial Hospital at Lin-Kou, Tao-Yuan, Taiwan; 2 Department of Plastic & Reconstructive Surgery, Chang Gung Memorial Hospital at Lin-Kou, Tao-Yuan, Taiwan; 3 Department of Radiation Oncology, Chang Gung Memorial Hospital at Lin-Kou, Tao-Yuan, Taiwan; 4 Department of Internal Medicine, Division of Hematology & Oncology, Chang Gung Memorial Hospital at Lin-Kou, Tao-Yuan, Taiwan; 5 Molecular Medicine Research Center, Chang Gung University, Tao-Yuan, Taiwan; 6 College of Medicine, Chang Gung University, Tao-Yuan, Taiwan; Karolinska Institutet, Sweden

## Abstract

**Objectives:**

The aim of this cohort study was to examine the role of the chemokine (C-X-C motif) ligand 9 (CXCL9) on nasopharyngeal carcinoma (NPC).

**Materials & Methods:**

Sera from 205 NPC patients and 231 healthy individuals, and 86 NPC tumor samples were enrolled. CXCL9 expression in tissue samples was analyzed by quantitative real-time PCR and immunohistochemistry. CXCL9 serum concentrations were measured by enzyme-linked immunosorbent assay.

**Results:**

CXCL9 expression was significantly higher in tumors than in normal epithelium. CXCL9 serum concentrations were also significantly higher in NPC patients compared to those in healthy individuals (516.8±617.6 vs. 170.7±375.0 pg/mL, *P*<0.0001). Serum CXCL9 levels were significantly higher in NPC patients with higher tumor stages, nodal stages, and overall stages (*P*<0.001, *P* = 0.001, and *P*<0.001, respectively). We found a statistically significant correlation between the concentrations of CXCL9 and EBV DNA load in the NPC patients (Spearman’s correlation analysis; *r* = 0.473, *P*<0.001; 95% confidence interval, 0.346–0.582). Moreover, NPC patients with higher CXCL9 levels (>290 pg/mL, median) before treatment had worse prognoses for overall survival and disease-free survival (*P* = 0.045 and *P* = 0.008, respectively). Multivariate logistic regression analyses also indicated that higher CXCL9 serum levels were an independent prognostic factor for disease-free survival (*P* = 0.015).

**Conclusion:**

Our study demonstrated that CXCL9 is associated with tumor burden and aggressiveness of NPC tumors and the serum level of this ligand may be useful as a prognostic indicator.

## Introduction

Nasopharyngeal carcinoma (NPC) is a rare head and neck malignancy worldwide, except for certain endemic areas in Southeast Asia including southern China, Hong Kong, and Taiwan [1]. Except for the distinct geographic and ethnic distribution, Epstein-Barr virus (EBV) is also known to be closely related to the carcinogenesis of NPC [2]. Therefore, various EBV-derived markers, such as EBV-specific viral capsid antigen (VCA) IgA and circulating cell-free EBV DNA, have been discovered as tumor markers for NPC [3], [4]. Despite the advances of radiotherapy and chemotherapy, some patients in the endemic areas still fail locoregionally or distantly under contemporary treatment. Thus the development of novel tumor markers stratifying the treatment outcomes might contribute to better prediction of prognosis and more insights with regard to the mechanisms of treatment failure.

In addition to EBV-related tumor markers, the role of chemokines in the NPC tumor cells has also been being studied [5], [6]. Chemokines represent a large family of proteins characterized by structural homologies based on conserved cysteine residues; four families of chemokines have been described according to the relative position of the conserved cysteine residues: CC, CXC, XC, and CX3C [7]. These molecules can work as tumor growth factors that relate to proliferation and angiogenesis, promoting tumor cell chemotaxis and contributing to tumor progression and even distant metastasis [8], [9]. We speculated that levels of the tumor-related cytokines would also be dysregulated in the tumor microenvironment and/or systemic circulation and associated with the growth of NPC. In a previous study, we have used the multiplex suspension array system, a high-throughput proteomic platform, to measure 48 different cytokines/chemokines simultaneously and discovered that circulating plasma chemokine (C-X-C motif) ligand 9 (CXCL9) levels were significantly elevated in the patients with oral cavity squamous cell carcinoma and nasopharyngeal carcinoma [10], [11]. However, the clinical roles and applications of CXCL9 serum levels in NPC patients still remain unclear.

In the current study, we initially confirmed that CXCL9 overexpression in NPC tumors by both quantitative real-time RT-PCR and immunohistochemistry. Next, the enzyme-linked immunosorbent (ELISA) method was used to measure the CXCL9 serum levels and evaluated the potential clinicopathologic relevance and role as a prognostic indicator in our NPC patients. The correlation between CXCL9 serum levels and EBV DNA load was also analyzed to clarify its relationship with the NPC tumor burden. Finally, we examined the survival analyses with CXCL9 serum levels and several possible prognostic factors to determine the potential role of CXCL9 serum levels as a prognostic indicator for NPC.

## Materials & Methods

### Patients, Clinical Staging Protocol, Oncologic Treatment, and Clinical Outcome Assessment

This study was approved by the ethics committee of Chang Gung Memorial Hospital – Linkou Medical Center. All subjects signed an Institutional Review Board-approved informed consent prior to study participation. The serum samples were obtained from 205 consecutively enrolled and newly identified patients with NPC tumors and 231 healthy individuals seen in Chang Gung Memorial Hospital, Linkou, from August 2003 to November 2009. The tumor tissue samples for immunohistochemical analyses were obtained from 86 patients with untreated NPC tumors during the same period (Table 1). The inclusion and exclusion criteria in the study were detailed in our previous report [11]–[13]. The TNM stage was defined according to the 2002 cancer staging system revised by the American Joint Committee on Cancer (AJCC) [14]. All NPC patients had been biopsy-proven and had undergone routine check-ups including head and neck magnetic resonance imaging, chest x-rays, abdominal ultrasonographies and bone scans, before treatment and every 6 months after treatment, according to the standard protocol. Fluorodeoxyglucose-positron emission tomography (FDG-PET) scans were performed in all untreated NPC patients to confirm the initial tumor stage. The control subjects were all volunteers undergoing routine health examinations or individuals presenting with otolaryngological-related, non-neoplastic diseases. Patients and controls with histories of malignant disease were excluded from the study. Blood samples were collected before treatment and during regular follow-ups.

**Table 1:** Characteristics of NPC patients and control subjects.

Characteristics	Patient number		Control serum number	
	Tumor tissues	Serum samples		
Gender				
Male	56	142	187	
Female	30	63	24	
Age (years)				
mean±SD	47.2±12.4	48.7±13.6	49.2±14.5	
Histological type^a^				
UC	49	123		
NKC	35	77		
SCC	2	5		
Overall pathological stage				
Stage I	5	13		
Stage II	23	46		
Stage III	31	74		
Stage IV	27	72		
Total	86	205		231

a UC, undifferentiated carcinoma; NKC, non-keratinizing carcinoma; SCC, squamous cell carcinoma.

All patients enrolled in the prospective cohort had been treated with definitive radiotherapy (cumulative dose of external beam radiotherapy ≧64.8 Gy). According to our current NPC treatment protocol, all patients whose tumor stages were equal to or exceeded 3 received additional cisplatin-based concurrent chemoradiotherapy in the Department of Radiation Oncology at CGMH [15], [16]. Patients who were diagnosed with distant metastatic disease at presentation (M1 stage), and/or who had undergone previous treatment at other institutes were excluded from the present study. Patients were followed-up at 3-month interval during the first 3 years after therapy and at 6-month interval thereafter.

### Quantitative Real-time RT-PCR

Sixteen paired NPC tumor and pericancerous normal tissues were homogenized in liquid nitrogen with a mortar and pestle, incubated with RNAzol B reagent (Tel-Test, Friendwood, TX), and total RNA was extracted according to the manufacturer’s protocol. The RNA was further purified using an RNeasy cleanup kit (Qiagen, Valencia, CA) according to the manufacturer’s protocol. First-strand cDNA was synthesized from 5 µg of total RNA and then mixed with a reaction mixture consisting of commercially available primers (CXCL9, Hs00171065_m1 and normalization control GAPDH, Hs99999905_m1; Assay-on-Demand, Applied Biosystems, Foster City, CA), RNase-free water, and TaqMan Universal PCR Master Mix. Quantitative real-time RT-PCR was performed and analyzed using a 7900 HT Sequence Detection System and SDS version 2 (Applied Biosystems, Foster City, CA), respectively. All experiments were repeated in triplicate, and the mean fold-change was calculated for each sample.

### Detection of the Presence of EBV LMP-1 Gene in the Specimens

We chose to amplify the regions in the EBV latent membrane protein-1 (LMP-1) gene as EBV detection markers in NPC samples. The procedures of DNA extraction from formalin-fixed paraffin-embedded samples, PCR amplification, and gel electrophoresis were described as we reported previously [17]. For PCR amplification, oligonucleotide primers for detecting the LMP-1 gene (sense BN1∶5′-AGC GAC TCT GCT GGA AAT GAT-3′ or antisense BN2∶5′-TGA TTA GCT AAG GCA TTC CCA-3′) were used. Negative control samples containing water were always processed in parallel to the patient samples. DNA from the B95.8 cell line was employed as the EBV positive control.

### Immunohistochemistry

Consecutive slide-mounted NPC sections were first treated with proteinase K at room temperature for 15 minutes. Endogenous peroxidase activity was inhibited by incubating with 3% H_2_O_2_ (DAKO, Glostrup, Denmark). Nonspecific binding was blocked with Antibody Diluent and Background Reducing Component (DAKO, Glostrup, Denmark). Sections were then incubated with anti-CXCL9 (1∶20; R&D Systems, Minneapolis, MN) and anti-LMP1 (1∶20; DAKO, Glostrup, Denmark) antibodies at room temperature for 1 hour. After a washing step, a HRP-conjugated secondary antibody was added and sections were incubated at room temperature for 20 minutes. Tissue sections were then treated with DAB reagent (DAKO, Glostrup, Denmark); 3,3′-diaminobenzidine tetrahydrochloride was used as a chromogen. Images of the stained slides were obtained using a ScanScope CT automated slide-scanning system (Aperio Technologies, Vista, CA). Expressions of CXCL9 and LMP-1 were scored using a combined scoring method that accounted for both the staining intensity and the percentage of stained cells [11], [12], [18]. Strong, moderate, weak, and negative staining intensities were scored as 3, 2, 1, and 0, respectively. For each of the intensity scores, the percentage of cells that stained at that specific level was visually estimated. The resulting combined score was calculated as the sum of the percentage of stained cells multiplied by the intensity scores. All specimens were independently evaluated by a pathologist (Liang Y.) without prior knowledge of the clinical origin of the specimen.

### ELISA Measurement

CXCL9 levels in the tested samples were determined using the ELISA kit Quantikine® for human CXCL9 (R&D Systems Minneapolis, MN). Human recombinant CXCL9 was used as the standard. Briefly, 100 µl of serum samples or standard were added to microtiter plates coated with a murine monoclonal antibody against human CXCL9 and incubated for 2 hours at room temperature. The plates were then washed three times with wash buffer, a horseradish peroxidase-conjugated polyclonal antibody was added to the wells, and the plates were incubated for 2 hours at room temperature. The plates were then washed, and hydrogen peroxide and tetramethylbenzidine were added for color development at room temperature for 30 minutes. The reaction was stopped by addition of 2 N sulfuric acid, and the color intensity in each well was measured as the optical density using a microplate reader set to 450 nm. Each experiment was performed in duplicate.

### Quantitation of Plasma EBV DNA Load

DNA extraction and quantitation of the EBV DNA load in plasma were performed according to the previously described protocols [4], [12].

### Statistical Analysis

All statistical data are expressed as mean ± SD. A Wilcoxon test was used to examine significance. Spearman’s (nonparametric) rank correlation was calculated to measure the correlation between the CXCL9 serum levels and plasma EBV DNA load in NPC patients. All NPC patients received follow-up evaluations at our outpatient clinic until June 2012 or until their death. Kaplan-Meier plots were used for survival analysis, with statistical significance measured by the log-rank test. Multivariate regression analyses were used to define specific risk factors for overall survival and disease-specific survival. A *P*-value <0.05 was considered statistically significant. All statistical analyses were performed using SAS software version 9.1 (SAS Institute Inc., Cary, NC).

## Results

### Overexpression of CXCL9 in Tumor Cells of NPC Tissues

**Figure 1:**
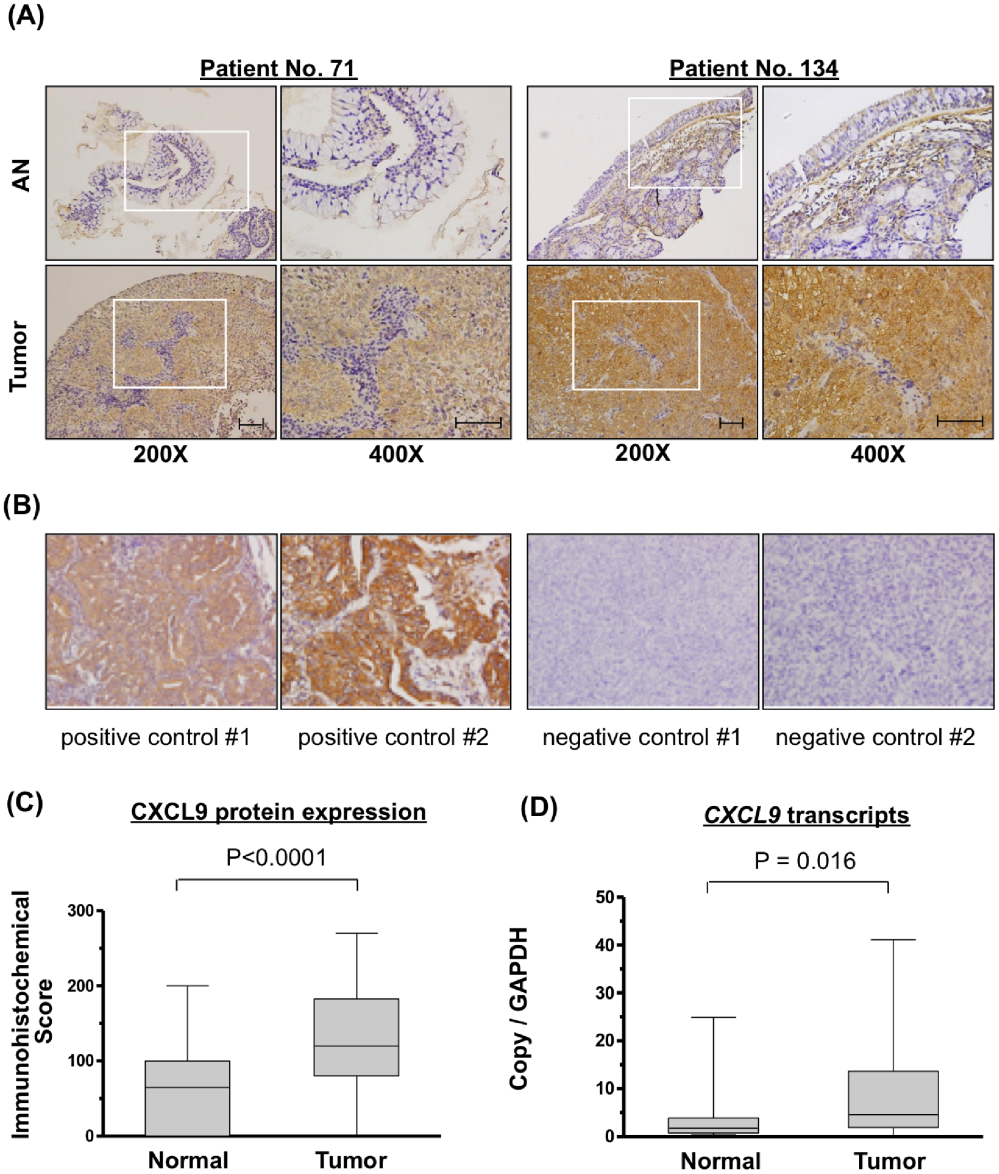
Overexpression of CXCL9 in NPC tissues. (A) Immunohistochemical staining of CXCL9 in paired pericancerous adjacent normal (AN) and tumor tissues from two representative cases (scale bar: 100 µm). Brown signals indicate the CXCL9 expression. Images in the box (left panel, 200x) were enlarged and shown in the right panel (400x). (B) Positive and negative controls for CXCL9 staining. (C) Box and whisker plots showing the immunohistochemical staining scores of CXCL9 in 40 paired AN and tumor tissues. (D) Box and whisker plots showing CXCL9 mRNA transcript levels in the 16 paired pericancerous AN and tumor tissues, as assessed by quantitative real-time RT-PCR. CXCL9 was highly overexpressed in both analyses for NPC tissues. Box, the range of the middle 50% of CXCL9 level; line inside box, median; whiskers, minimal to maximal levels.

To distinguish which cell types in the tumor mass expressed CXCL9, we performed immunohistochemical staining of tissue sections from 86 patients with NPC. CXCL9 was highly expressed in the cytoplasm of tumor cells, but was nearly absent from the tumor infiltrating lymphocytes (Figure 1A). Samples from lung cancer and NPC specimens not treated by primary antibody against CXCL9 were used as the positive and negative controls (Figure 1B). CXCL9 positive cells were found to be scattered throughout the tumor mass with the various degree of intratumor heterogeneity. Moreover, the paired adjacent normal epithelium samples showed minimal or no expression of CXCL9 (Figure 1A). Statistical analysis of the 40 paired samples available from these 86 patients demonstrated that CXCL9 expression (score) was significantly higher in tumor cells versus normal epithelial cells (135.7±70.1 *vs.* 65.2±63.2, *P*<0.0001; Figure 1C). Transcripts for CXCL9 (fold-change) were significantly elevated in NPC tumor specimens as compared with adjacent normal tissue (8.2±10.3 *vs.* 4.3±6.5, *P* = 0.016; Figure 1D). Next, we evaluated the relationships between increased CXCL9 expression and various clinicopathological characteristics of NPC patients. However, we observed no association between CXCL9 overexpression in the immunohistochemical scores of NPC tumors and patient age, sex, clinical TNM stages or histopathological classifications (Table 2).

**Table 2:** Association of CXCL9 scores in immunohistochemistry with clinicopathological characteristics in 86 untreated NPC patients.

		CXCL9 scores	
	No.	(Mean ± SD)	P-value
Gender			
Female	30	134±71	0.630
Male	56	136±70	
Tumor stage			
I–II	46	124±68	0.066
III–IV	40	148±71	
Nodal Stage			
= 0, 1	46	140±69	0.651
= 2, 3	40	130±71	
Overall stage			
I–II	28	129±64	0.418
III–IV	58	138±73	
Differentiation*			
NKC	35	140±64	0.789
UC	49	135±71	

* UC, undifferentiated carcinoma; NKC, non-keratinizing carcinoma.

### EBV Status in NPC Specimens

**Figure 2:**
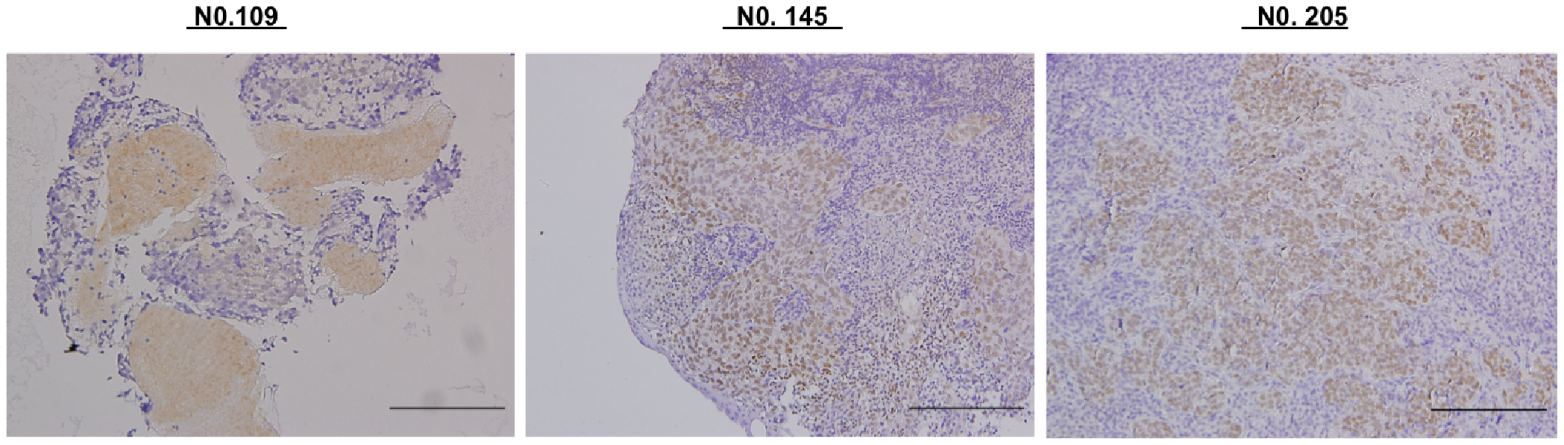
Immunohistochemical stainings of EBV LMP-1 in the NPC tumor tissues from 3 representative cases (scale bar = 200 µm). The LMP-1 immunohistological scores were 220, 200 and 100 for specimen No. 109, 145 and 205, respectively.

We extracted the DNA from available archival formalin-fixed paraffin-embedded samples (*n* = 122) and performed the PCR analyses for detecting EBV LMP-1 as we previously reported [17]. We discovered that EBV genome (LMP-1 gene) was positive in 113 samples (92.6%) in non-keratinzing cases of our NPC patients. Next, the same specimens subjected to immunohistochemistry for CXCL9 were subjected to immunohistochemistry for LMP-1 and we found that the EBV LMP-1 scores ranged from 0 (*n* = 32) to 220 (mean, 50; Figure 2). By correlation analysis of the immunohistochemical scores between CXCL9 and EBV LMP-1, we found a statistically significant correlation in these 86 NPC samples (*r* = 0.273, *P* = 0.011; 95% confidence interval, 0.063–0.460).

### Profiles of CXCL9 Serum Concentrations in Study Subjects

**Figure 3:**
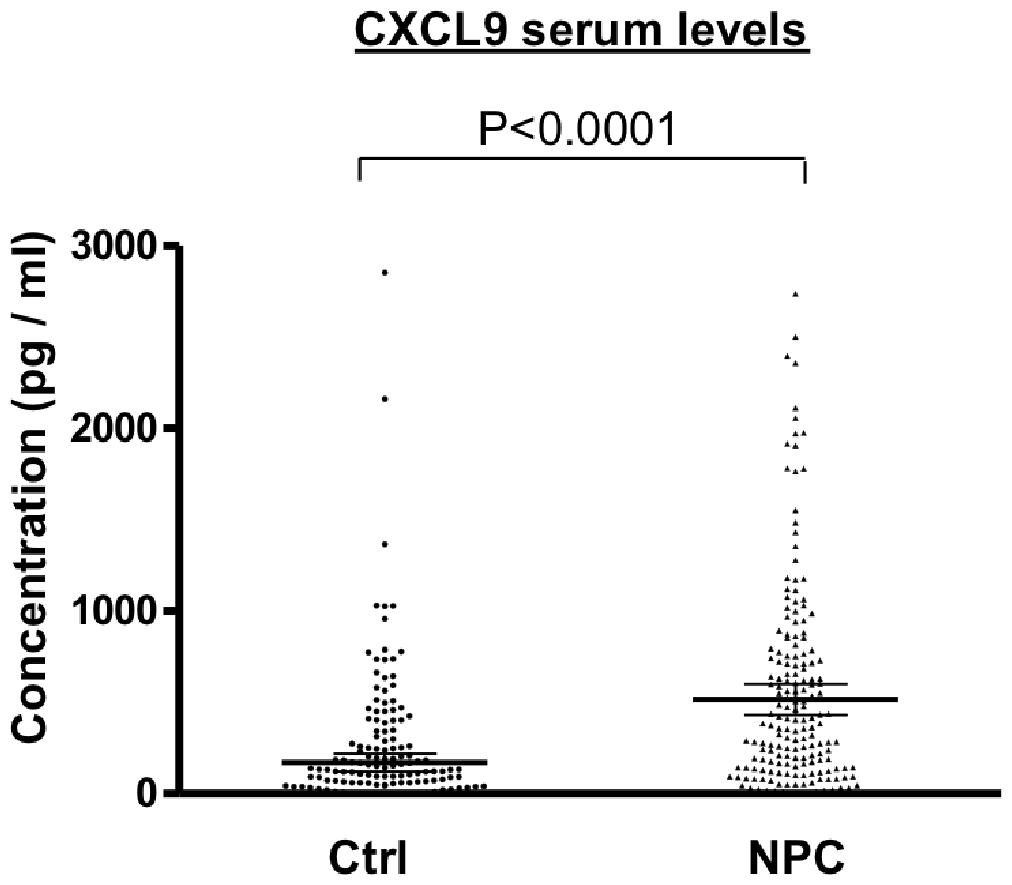
Elevated serum levels of CXCL9 and EBV DNA in NPC Patients. Serum levels of CXCL9 were measured in 205 pre-treated NPC patients (NPC) and 231 healthy controls (Ctrl).

The CXCL9 serum levels were found to be significantly different between patients with NPC and healthy individuals (516.8±617.6 pg/mL vs. 170.7±375.0 pg/mL, *P*<0.0001; Figure 3). Pretreatment CXCL9 serum concentrations were significantly higher in patients with higher tumor stages, nodal stages, and overall stages (*P*<0.001, *P* = 0.001, and *P*<0.001, respectively; Table 3). However, CXCL9 serum concentrations in the NPC patients prior to treatment were not significantly associated with gender or histopathological classifications (Table 3).

**Table 3:** Association of serum CXCL9 levels with clinicopathological characteristics in 205 untreated NPC patients.

		CXCL9 serum level	
	No.	(Mean ± SD, pg/mL)	P-value
Gender			
Female	63	424.2±545.8	0.300
Male	142	557.8±644.4	
Tumor stage			
I–II	105	390.4±523.7	<0.001†
III–IV	100	613.3±642.7	
Nodal Stage			
= 0, 1	103	336.9±474.4	0.001†
= 2, 3	102	656.8±655.3	
Overall stage			
I–II	59	212.0±277.4	<0.001†
III–IV	146	617.2±674.6	
Differentiation*			
NKC	77	512.1±293.5	0.897
UC	123	497.2±585.5	

* UC, undifferentiated carcinoma; NKC, non-keratinizing carcinoma.

† statistically significant.

### Correlation between Serum Concentrations of CXCL9 and EBV DNA Load

Because the EBV DNA load before treatment has been recognized as a marker of tumor burden and disease stage (T and N stage) as quantified by MRI and FDG-PET [19], [20], the correlation of concentrations between the CXCL9 and EBV DNA load in circulation was also analyzed to investigate their potential association with NPC tumor burden and EBV. By Spearman’s correlation analysis between the concentrations of CXCL9 and EBV DNA load, we found a statistically significant correlation between the concentrations of CXCL9 and EBV DNA load in the NPC patients (Spearman’s correlation analysis; *r* = 0.473, *P*<0.001; 95% confidence interval, 0.346–0.582).

### Correlation of CXCL9 Serum Levels with Patients’ Overall Survival (OS) and Disease-free Survival (DFS)

**Figure 4:**
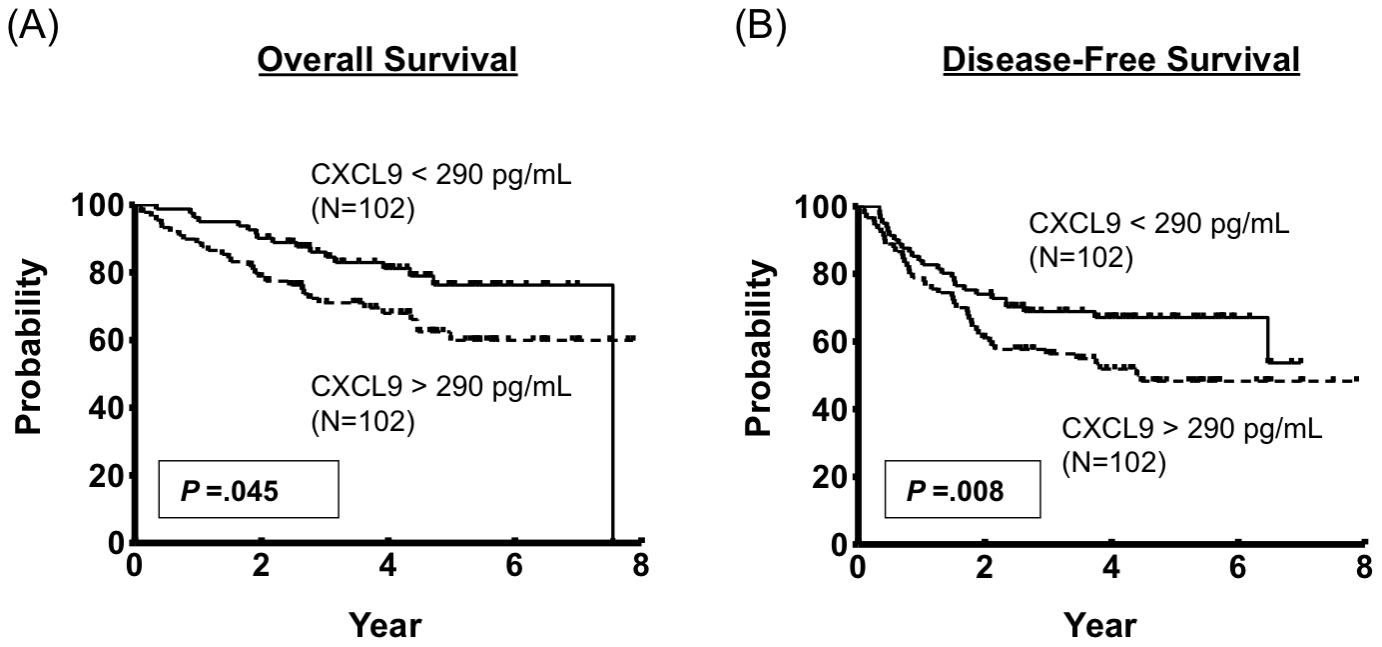
Association of higher CXCL9 serum levels with a poorer prognosis of NPC patient overall survival (OS) and disease-free survival (DFS).

To evaluate whether the CXCL9 serum levels were associated with patient survival, we analyzed 205 consecutive NPC patients enrolled in the study after the treatment. Five-year OS for patient subgroups stratified by the higher (*N* = 102) vs. lower (*N* = 102) levels of serum CXCL9 (290 pg/mL, median level) were 59.9% vs. 76.2% when compared using a log-rank test (*P* = 0.045, Figure 4A). Similarly, NPC patient subgroups stratified by the higher vs. lower levels of serum CXCL9 had significantly different five-year DFS rates of 48.3% vs. 67.1%, respectively (*P* = 0.008, Figure 4B). To determine whether higher CXCL9 serum levels was an independent predictor of DFS, a multivariate analysis was performed with age, gender, overall stage, EBV DNA load, and CXCL9 serum levels as parameters. Only CXCL9 serum concentrations were independent predictors of DFS (*P* = 0.015; Table 4), but the remaining factors (age, gender, overall stage, EBV DNA load) were not. These results indicated that CXCL9 serum concentrations could be useful in predicting prognosis in NPC patients after treatment.

**Table 4:** Cox proportional hazard models on disease-free survival of NPC patients.

	Multivariatedjusted HR (95% CI)^a^	P-value
Age^b^		
>45.3 vs. <45.3	1.231 (0.598–2.531)	0.572
Gender		
Male vs. Female	1.342 (0.540–3.335)	0.527
Overall Stage		
3, 4 vs. 1, 2	0.938 (0.358–2.453)	0.895
EBV DNA load		
High vs. low^c^	1.015 (0.471–2.189)	0.968
Serum CXCL9 level		
High vs. Low^c^	2.972 (1.227–7.199)	0.015†

† statistically significant;

a HR: Hazard Ratio; CI: Confidence Interval; also adjusted with sex and gender;

b median age (year);

c Cut-off value: 307 copy/mL and 290 pg/mL, respectively (median values).

## Discussion

We previously demonstrated that circulating plasma CXCL9 levels were differentially elevated in the both groups of patients with oral cavity squamous cell carcinoma and nasopharyngeal carcinoma by the multiplex suspension array system, a high-throughput proteomic platform [10]. However, no other study has been conducted to clarify the association between NPC and CXCL9 and the potential roles of CXCL9 in NPC patients still remain unclear. This study is the first to confirm the CXCL9 overexpression in mRNA and protein levels of NPC. To the best of our knowledge, although one previous study reported the detection of CXCL9 transcript in some head-and-neck cell lines [21], no other study has ever addressed the CXCL9 expression in NPC. Similarly, the prognostic value of CXCL9 serum levels for patients with NPC tumors in this study also has not been demonstrated previously. This study discovered that CXCL9 serum levels were statistically higher in patients with NPC compared to those of healthy controls. In addition, the higher CXCL9 serum levels were associated with pretreatment tumor stages, nodal stages, and overall stages. Moreover, higher CXCL9 serum levels of NPC patients were significantly associated with OS and DFS. Finally, the serum CXCL9 level had a statistically significant positive correlation to that of EBV DNA load. These results indicated that CXCL9 is over-expressed in NPC cells and circulating CXCL9 levels were associated with NPC tumor burden and progression. CXCL9 levels affected disease-free and overall survival in our NPC patient cohort.

CXCL9 is an interferon gamma-inducible chemokine secreted by macrophages and neutrophils, which works as a potent chemoattractant for activated T cells and also plays an important role in the acute rejection response during organ transplantation [22], [23]. From the relationship upon tissue inflammatory reaction, its anti-tumor effect was originally believed to originate from the chemotactic functions that resulted in increased T lymphocyte infiltration and play a critical role in inhibition of local tumor growth for malignant melanoma [24]. In addition, the coupling of CXCL9 with its receptor CXCR3, has anti-angiogenic properties through direct interaction with the endothelium, causing suppression of tumor growth due to the suppression of neovascularization [9]. However, other studies showed that CXCL9 expression also could be seen during active tumor growth. High expressions of CXCL9, along with CXCR3, were seen in thyroid and gastric marginal zone lymphoma and also mucosa associated lymphoid tissue type lymphoma, which were speculated to be associated with autocrine function and the migration of lymphoma cells [25]. In the role of early cancer detection, the elevated CXCL9 concentrations in blood samples of patients with early breast cancers (compared to those of normal volunteers) indicated that CXCL9 could be a screening blood marker for breast cancers [26]. A scoring method, comprising a combination of Fibronectin1 and CXCL9 serum concentrations, has shown a remarkable increase in detection (up to 54%) from ER-negative cases in breast cancer [26].

Since the pathogenesis of NPC closely correlates with EBV infection and subsequent T-cell infiltration, several studies also investigated other chemokine/chemokine receptor expression in NPC [27]–[29]. Understanding the roles of chemokines in NPC tumors could contribute to identifying the tumor-homing phenotypes with the potential T-cell based therapies and the development of chemokine receptor antagonists for NPC treatment [6]. In our previous study, serum levels of CCL20 were demonstrated to indicate the disease status and be a prognosticator for NPC. Additionally, CCL20 contributed to the migration and invasion of NPC cells [12]. Parsonage et al. [6] reported the observation with respect to the enrichment of T-cell expressing CXCR6 and CCR5 at NPC tumor site were and the expression of CXCR6 being strongly related to infiltration of regulatory T cells that promote tumor growth. Lu et al. [5] also used the ELISA method to measure the serum levels of CCL2 and TNF-α among NPC patient and showed that a significantly lower 5-year post-treated survival found in the group with low serum levels for both biomarkers. Lai et al. [30] addressed that the NPC cells expressing the endogenous latent membrane protein 1 could enhance the chemoattraction to primary T cells that contributes to the pathogenesis of malignancy. Although a previous study demonstrated that CD8(+) T cells were found to represent the majority of tumor infiltrating leukocytes in primary brain tumor and tend to accumulate in perivascular areas. Their density was found to be correlated with the expression of CXCL9 in the perivascular microenvironment [31]. However, in NPC, the potential correlation between the level of CXCL9 expression by NPC tumor cells and the extent of T cell infiltrate still awaits further investigation.

The current study demonstrated the overexpression of CXCL9 on the NPC cells occurred mainly from the cytoplasm but was nearly absent from the infiltrating lymphocytes or normal tissue cells in the adjacent tissues. This observation demonstrated that the expression of CXCL9 was highly related to the autocrine or paracrine regulation of NPC tumor cells. Another novel finding in the current study is that the serum CXCL9 levels were significantly correlated to the EBV DNA load in NPC patients before treatment. It is well established that EBV DNA load in the circulation is positively correlated with the tumor volume and its metabolic activity in NPC tumors, indicating its current role in the clinical application as a blood marker for prognostic prediction and post-treatment monitoring [4], [19]. Therefore, the serum CXCL9 levels could potentially be used as an effective blood marker for NPC patients, although further investigation in another prospective cohort is needed to demonstrate clinical utility. Furthermore, the prognostic value of serum CXCL9 was also observed in our NPC patients by analyzing the survival from patient groups dichotomized by high and low serum levels of CXCL9. Both OS and DFS were significantly lower in the group with high serum levels of CXCL9. The multivariate analysis showed the pre-treatment CXCL9 serum level was also an independent prognostic predictor of DFS, indicating its potential role as an effective prognosticator in contemporary NPC treatment. The findings in the current study also support the fundamental importance of the CXCL9 pathway in NPC tumors. CXCL9 serum levels might be considered as a therapeutic marker for personalized treatment if CXCL9/CXCR3 blockade therapy is proved to be a useful adjunctive treatment for NPC in the future. Additionally, more in-vivo experiments and clinical trials are warranted to evaluate the role of CXCL9 for detecting tumor recurrence or distant metastasis in the future.
